# Current Perspectives on Taxanes: Focus on Their Bioactivity, Delivery and Combination Therapy

**DOI:** 10.3390/plants10030569

**Published:** 2021-03-17

**Authors:** Jan Škubník, Vladimíra Pavlíčková, Tomáš Ruml, Silvie Rimpelová

**Affiliations:** Department of Biochemistry and Microbiology, University of Chemistry and Technology Prague, Technická 3, 166 28 Prague 6, Czech Republic; jan.skubnik@vscht.cz (J.Š.); vladimira.pavlickova@vscht.cz (V.P.); tomas.ruml@vscht.cz (T.R.)

**Keywords:** anticancer effect, antimitotic agents, cancer treatment, combination therapy, docetaxel, microtubule-stabilizing agents, natural products, paclitaxel, taxanes, targeted drug delivery

## Abstract

Taxanes, mainly paclitaxel and docetaxel, the microtubule stabilizers, have been well known for being the first-line therapy for breast cancer for more than the last thirty years. Moreover, they have been also used for the treatment of ovarian, hormone-refractory prostate, head and neck, and non-small cell lung carcinomas. Even though paclitaxel and docetaxel significantly enhance the overall survival rate of cancer patients, there are some limitations of their use, such as very poor water solubility and the occurrence of severe side effects. However, this is what pushes the research on these microtubule-stabilizing agents further and yields novel taxane derivatives with significantly improved properties. Therefore, this review article brings recent advances reported in taxane research mainly in the last two years. We focused especially on recent methods of taxane isolation, their mechanism of action, development of their novel derivatives, formulations, and improved tumor-targeted drug delivery. Since cancer cell chemoresistance can be an unsurpassable hurdle in taxane administration, a significant part of this review article has been also devoted to combination therapy of taxanes in cancer treatment. Last but not least, we summarize ongoing clinical trials on these compounds and bring a perspective of advancements in this field.

## 1. Introduction

**Figure 1:**
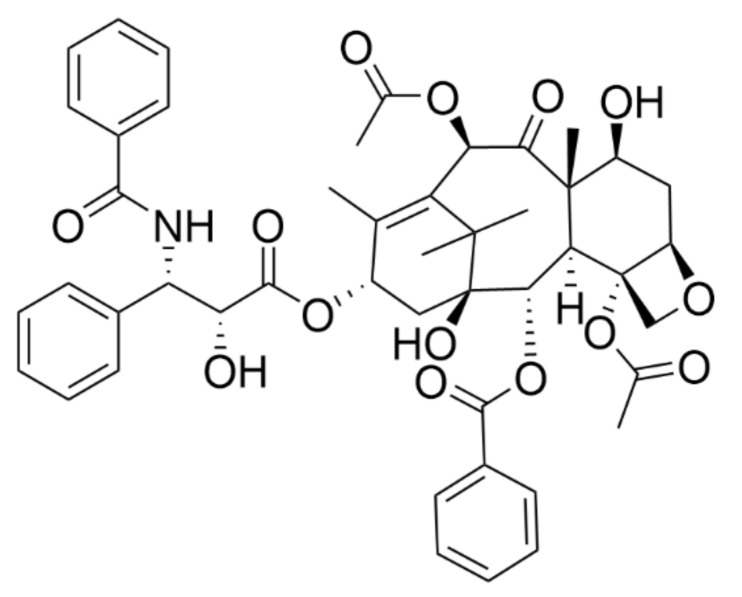
Chemical structure of paclitaxel.

Cancer represents a long-time burden and, despite great progress in the knowledge of this disease, a detailed understanding of it is still lacking, which reduces the success of the treatment of more serious cases. Still, we seek novel treatment approaches, such as personalized medicine, and novel drugs. There are many opportunities to obtain new powerful and specific drugs, i.e., repositioning of substances already approved for other indications, searching for completely new compounds, or improving the properties of current anticancer drugs used in clinics. In the combat against cancer, natural compounds play a principal role. These are mainly plant secondary metabolites among which taxanes are the most massively used ones. Taxanes are natural diterpenoid substances occurring in yew plants, which received their name from a Latin term for yew, *Taxus sp*. The best known and to date the most clinically used taxane is paclitaxel (Figure 1). Paclitaxel was originally isolated in the 1960s from the stem bark of the western yew, *Taxus brevifolia,* and its structure was characterized in 1971 by Wani et al. [1]. Nowadays, paclitaxel is marketed as Taxol^®^ and it was not approved for clinical use until 1994; the first medicinal application was ovarian cancer treatment [2].

**Figure 2:**
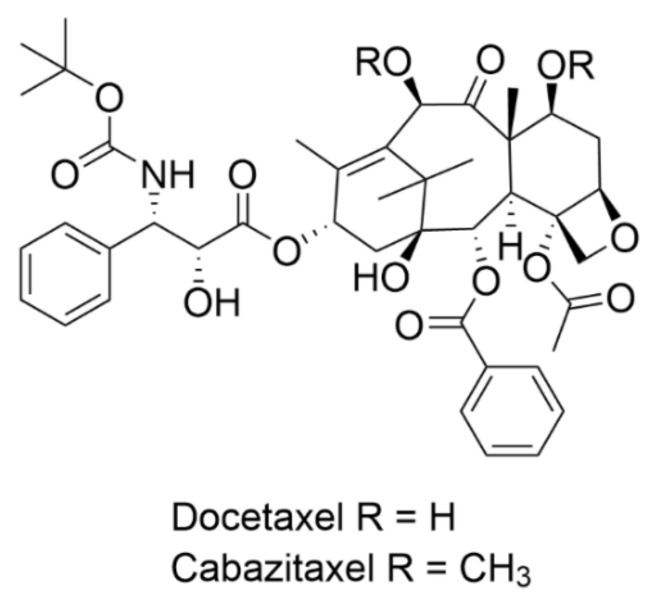
Chemical structure of docetaxel and cabazitaxel.

After the discovery of the unique mechanism of action of paclitaxel, which is tubulin binding and enhanced microtubule polymerization, a great effort has been made to further study taxanes and produce their synthetic and semisynthetic analogues with enhanced properties and improved water solubility. The most successful semisynthetic analogue of paclitaxel is docetaxel (Figure 2) marketed as Taxotere^®^. It was first approved by the European Medicines Agency (EMA) in 1995, which was followed by the U.S. Food and Drug Administration (FDA) authorization in 1996 for the treatment of breast cancer [3]. Another clinically used semisynthetic taxane is cabazitaxel (Jevtana^®^), which was approved by the FDA in 2010 for prostate cancer treatment [4].

Even though cancer therapy research has been continuously emerging, taxanes remain the first-line therapy for breast cancer and one of the most commonly used types of chemotherapy for other types of cancer. Nevertheless, soon after the clinical approval of taxanes and their massive administration in clinical practice, some drawbacks of these remedies emerged. These were mainly their poor water solubility and the more and more frequently occurring resistance of tumor cells to treatment with taxanes. This initiated a search for novel taxane formulations with improved water solubility and delivery to tumors. Moreover, combinations of taxanes with various other agents are being tested to overcome frequent chemoresistances and to increase the anticancer activity via a synergistic effect.

Except for the aforementioned limitations of taxane administration, another big issue waiting to be solved relates to the limited sources of taxanes and their complicated production. The sources for taxane isolation are not only very scarce but the production process is also ecologically very demanding. Hence, lately, attention has been focused on the understanding of taxane biosynthesis, since it could reveal novel options for commercially profitable taxane production.

Therefore, this review article aims to show the up-to-date state of the art on taxane research, to cover the approaches to their isolation, synthesis, biosynthesis, improved solubility, and tumor targeting specificity, and also to discuss the current knowledge on their mechanism of action and development of cancer cell chemoresistance. The greatest part of this review article is dedicated to the combination therapy of taxanes, as it is currently the leading line of this research topic.

### 1.1. Natural Sources of Paclitaxel and Isolation Processes

**Figure 3:**
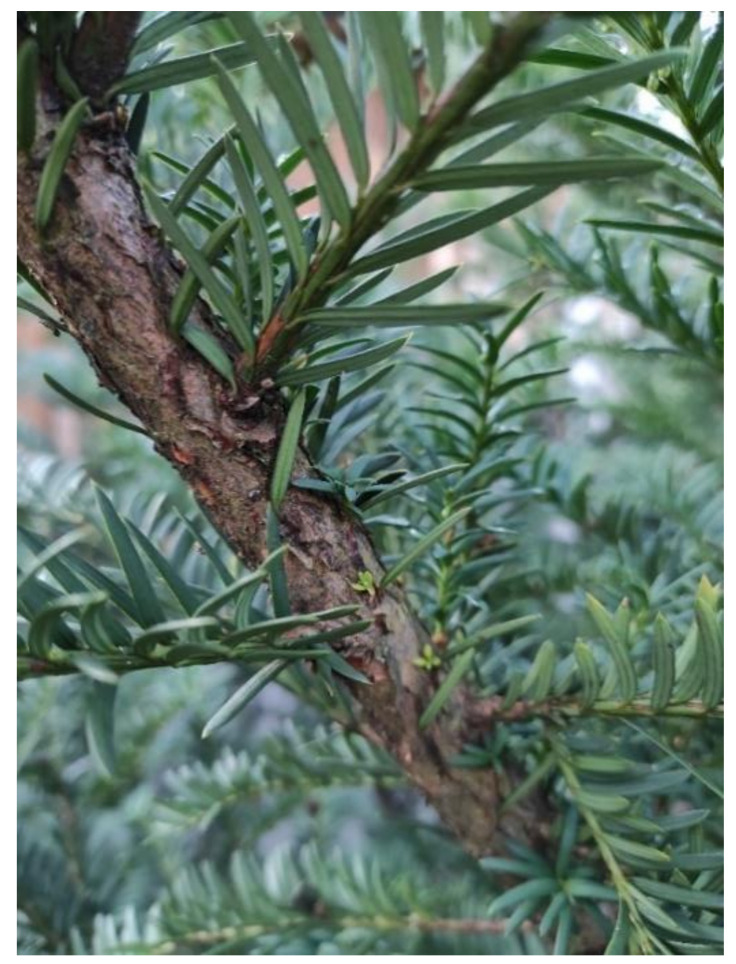
A branch of a yew; paclitaxel is isolated from the bark, needles, or whole branches of yew.

Taxanes occur naturally in a plethora of plants and microorganisms, but mainly they can be found in yews, *Taxus sp.* (*Taxaceae).* These are both monoecious and dioecious trees and shrubs with irregularly arranged branches and persistent needles, occurring mainly in the northern hemisphere. The genus *Taxus* contains 12 to 24 species (the number differs in particular publications), and as many as 55 variants of yew are distinguished [5,6]. Originally, paclitaxel was commercially isolated from the bark of *Taxus baccata*, the European yew tree (Figure 3), which contains the greatest amount of the compound among all kinds of yew. Isolation from yew was firstly used also commercially. Because bark harvesting is extremely non-ecological, nowadays, there are more feasible methods of taxane production, such as semisynthesis, see Section 1.2. However, this method involves also growing yew trees and harvesting the yew material. Thus, novel sources of paclitaxel are sought.

**Figure 4:**
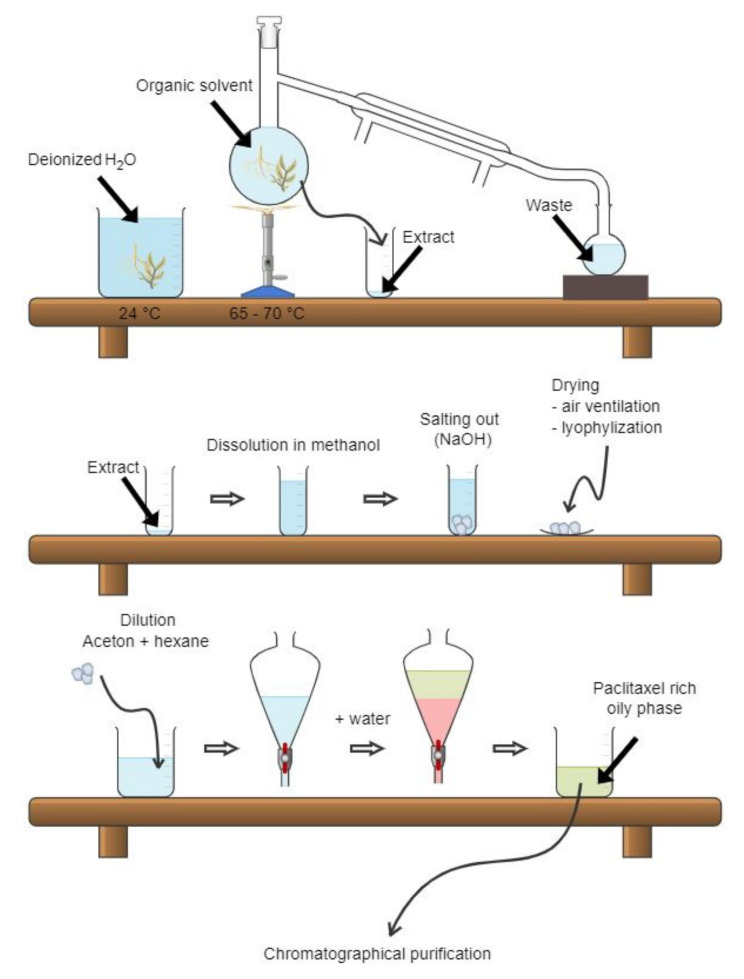
Scheme of basic paclitaxel isolation, created with Chemix software.

The basic paclitaxel isolation process was described only in a patent. We provide a summary of this process, though we are concerned about the quality of the protocol, since patents are not independently reviewed. The paclitaxel isolation process (Figure 4) begins with the rinsing of raw plant material, preferably bark, with deionized water for 3 h. After the water is removed, extraction into organic solvents (alcohols, ketones, or a mixture of both) follows. The extract is transferred into a tank and heated up to 65–70 °C, which results in the removal of the organic solvents by a distillation process. The residual solution is transferred into another tank, and the biomass is concentrated by salting out from the solution previously diluted with methanol and water. The precipitated biomass is then separated and dried by ventilation or lyophilization. To purify the obtained biomass, it is dissolved in the mixture of acetone and hexane and, subsequently, water is added into the solution to form the paclitaxel-rich oily phase. This phase is then chromatographically purified and the solution crystalized. This process, which can be repeated several times to achieve higher purity, comprises mixing of the oily-phase with a silica gel and drying by ventilation. The silica gel coated with the paclitaxel phase is then applied onto a chromatography column filled with the same type of silica gel to further purify the sample using an elution mixture of 35% acetone and 65% hexane. Then, the paclitaxel-containing fractions are combined, dried, and dissolved in pure acetone. Finally, paclitaxel is crystallized by adding hexane into the solution [7].

Great effort has been put into the optimization of this standard patented process for paclitaxel isolation. The least demanding approach is the optimization of the solvent composition. Considering the properties of commonly used organic solvents, the best option seems to be 100% acetone [8]. However, it is also possible to use atypical solvents or co-solvents, for example, ionic liquids. These solutions composed of cations and anions at room temperature possess certain advantages, such as low volatility and high chemical stability at atmospheric pressure. With the use of such liquids as co-solvents, much higher paclitaxel yields can be gained and the extraction time can be significantly reduced [9].

Besides solvent engineering, there are also other options to augment the overall efficacy of paclitaxel isolation. One of the possibilities is to use a so-called accelerated solvent extraction, which ensures higher paclitaxel yields and reduced extraction time. The basis of this method is increased pressure in an extraction cell to keep the solvent warm during the whole process [10]. A more recent and quite effective method is microwave-assisted extraction, during which the methanol-water solution of the organic material is irradiated with microwaves in a closed-vessel system. Such a method is advantageous mainly for its time efficiency. Instead of hours of extraction when using the standard method, this method allows complete extraction in one cycle within a few minutes [11]. However, the question remains whether it would be possible to utilize this method for commercial production of paclitaxel, since, to date, performed experiments were done only under laboratory conditions.

In the laboratory, it is possible to try diverse approaches to obtain taxanes, which do not necessarily include working with plant material. In the following paragraphs, we will describe other discovered production options. Unfortunately, the most commercially feasible one is still not a very eco-friendly option for paclitaxel semisynthesis. An alternative to avoid using plant material is taxane production in endophytic fungi. To date, dozens of fungal strains able to naturally produce taxanes *in vitro* have been reported, see review in ref. [12]. Recent advances in this production approach cover mainly optimizations of the paclitaxel fermentation conditions and involvement of novel techniques in fungal taxane production. An example of improvement of taxane systematic production was reported by El-Sayed et al. [13], who used the endophytic fungal strain of *Epicoccum nigrum* TXB502. In this study, first, cultivation conditions were optimized to increase paclitaxel production; then, a stable mutant of *E. nigrum* TXB502 was prepared by gamma irradiation, which enhanced paclitaxel yields. Finally, the authors prepared alginate-immobilized mycelia of the stable mutant of *E. nigrum* TXB502, yielding an approximately 22-fold higher taxane production compared to the natural strain. The most promising fungal strain in terms of paclitaxel production was reported by Kumar et al. [14], who isolated a Northern Himalayan endophytic *Aspergillus fumigatus* fungi which yielded 1.6 g of paclitaxel per liter of the fermentation medium. Such a high yield represents a high potential for this strain to be commercially used in the future. Since 1 kg of paclitaxel is gained from approximately 600 L of the fermented fungi, whereas to gain the same amount of the compound by isolation from yew, approximately 30,000 kg of the yew material is needed [15].

Besides optimizing fungal growth conditions, an effective approach leading to augmented paclitaxel yields from fungi might be also the use of certain elicitors or effectors of the cellular processes in fungi. For this purpose, Subban et al. [16] confirmed the efficacy of salicylic acid on *Pestaloptiosis microspora*, which is one of the fungal species most commonly known for taxane production. The salicylic acid added to the fungal suspension at 300 µM concentration increased the paclitaxel production 45 times. This effect is probably caused by the ability of salicylic acid to increase reactive oxygen species production and lipid peroxidation, both of which significantly stimulate expression of the gene coding the geranylgeranyl pyrophosphate synthase (EC2.5.1.1), which is capable of higher taxane production. Fungi are not the only microbial producers of paclitaxel. The general approach of bioengineering, mainly promoting the transcription of particular genes, is hardly applicable in the fungal production of taxanes because only a few taxane-synthetic genes have been described until now. The current knowledge in this field was recently extensively reviewed by Sabzehzari et al. [17]. One of the most recent discoveries in fungal taxane production was made by Balabhadrapatruni et al. [18], who described two genes of paclitaxel biosynthetic pathway in *Fusarium solani*, namely taxane 13α-hydroxylase and 10-deacetylbaccatin III-10-βO-acetyltransferase, which could serve to further manipulate the yields of paclitaxel in this fungus. 

Furthermore, paclitaxel-producing bacteria have been discovered. The method of how to isolate paclitaxel-producing endophytic bacteria from *Taxus sp.* and how to improve the paclitaxel production in these microorganisms was patented already in 1996 and 2000 by Pagé et al. [19,20]. Interestingly, no other work on the topic of bacterial production of taxanes followed until 2010. Ajikumar et al. [21] reported the possibility of producing paclitaxel precursors in *Escherichia coli* with a yield of 1 g per liter of fermented bacteria for paclitaxel precursor taxadiene. Five years later, the same research group also reported an interesting option of co-culturing of *E. coli* with *Saccharomyces cerevisiae*, where both organisms possessed only partial taxane synthetic pathways. The ability of production of the final taxane molecules in this consortium was reported; however, only with a 33 mg·L^−1^ yield [22]. The question is, thus, what benefit does such a method bring to the general taxane production issue? Surprisingly, despite the promising initial results of their publications, no other research on the topic has been published. Recently, however, Subramanian et al. [23] returned to this topic and described the isolation of taxane-producing bacteria (*Bacillus flexus*, *Bacillus licheniformis,* and *Oceanobacillus picturae*) from non-yew material, namely from different marine macroalgae. They also identified a biosynthetic pathway of the terpenoid core in the isolated bacteria. The lack of scientific evidence for paclitaxel production arises questions about the credibility of such research. Given also the lack of continuity between the already published reports, we must consider them rather as thoughts-provoking pivotal data. More than with bacterial production of paclitaxel, it is worth studying other production methods, which are currently gaining greater interest.

One of them is paclitaxel production in plant tissue cultures, which is already commercially realized. It represents one of the most significant directions in research on taxane large-scale production. Cultivation of *Taxus* cells *in vitro* is possible, though yields of paclitaxel from such cell culture are not sufficient for large-scale production, even when elicitors, such as methyl-jasmonate, are used [24]. Because genetic manipulations of *Taxus* cells are rather demanding, other paclitaxel-producing plant cells have been investigated for the same purpose. The most promising plant in this field is *Corylus avellana,* known as European filbert or hazel. Although the amount of paclitaxel in this plant is significantly lower than in *Taxus sp.*, cultivation of cells from this plant and their biotechnological engineering does not require so much effort [25]. Originally, the presence of taxanes in hazel was attributed to endophytic fungi, but later experiments conducted in an aseptic environment confirmed the ability of hazel cells to produce taxanes on their own. Most probably, the genes for taxane production were adopted by hazel by horizontal gene transfer from fungi. Many genes from the taxane biosynthesis pathway in hazel have been identified, which could be used in bioengineering and enhancing taxane production [26]. The most recent experiments in optimizing paclitaxel production conditions in hazel were reported by Salehi et al. [27], who tested the impact of whole fungal elicitors from *Camarosporomyces flavigenus* (cell extract and culture filtrate together) on taxane production in *Corylus* cell culture. These elicitors significantly increased the biosynthesis and secretion of paclitaxel. Different ratios of cell extracts and filtrates were tested using 2.5, 5, and 10% (*v*/*v*) amounts of elicitors, respectively; in all cases, an increase in paclitaxel production was detected. Nevertheless, the yield increase was only one to five-fold compared to unaffected control *Corylus* culture, which is too little, given the complicated preparation and overall work with the fungal elicitors. Nevertheless, paclitaxel production in plant cells *in vitro* remains a valid option to be further studied and extended.

### 1.2. Biosynthesis, Synthesis, and Semisynthesis of Taxanes

**Figure 5:**
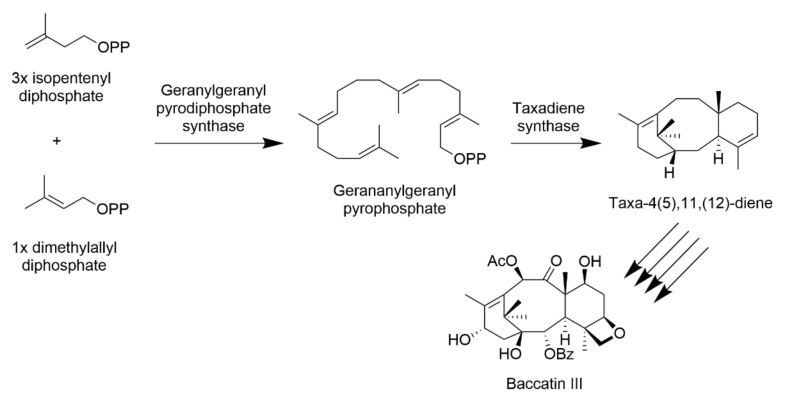
Simplified diterpenoid pathway of baccatin III synthesis.

**Figure 6:**
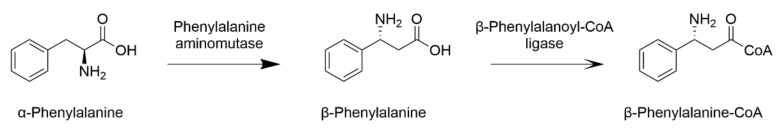
Simplified phenylpropanoid pathway of baccatin III synthesis.

To improve the overall yields of taxane production either in yew trees or in the other aforementioned systems, we need to understand the natural biosynthetic pathways and select the potential targets for bioengineering interventions. Paclitaxel and all taxanes are diterpenes; therefore, their biosynthesis similarly begins with all-natural diterpenoid compounds. Specifically, yew trees use two major metabolic pathways yielding paclitaxel, the diterpenoid pathway (Figure 5) and the phenylpropanoid pathway (Figure 6). The first one leads to the core of the taxane skeleton called baccatin III, while the second one provides a phenylisoserine side chain, which is added to the baccatin III structure. The precursors for taxane biosynthesis are isoprenoid structures isopentenyl diphosphate and dimethylallyl diphosphate. They are produced in two different, but yet in particular connected, pathways: the classic mevalonate pathway, which takes place in the cytosol, and the 2-C-methyl-d-erythritol-4-phosphate pathway occurring in plastids [26].

The starting compound for taxane biosynthesis is geranylgeranyl pyrophosphate (GGPP), which is synthesized from three isopentenyl diphosphate units and one molecule of dimethylallyl diphosphate by geranylgeranyl pyrophosphate synthase (EC 2.5.1.1). In the cyclase phase, GGPP then cyclizes to form the first compound of the diterpenoid pathway with the taxane key motif, taxadiene (taxa-4(5),11,(12)-diene). This key reaction is catalyzed by taxadiene synthase (EC 4.2.3.17). Briefly, in the oxidation phase, taxadiene undergoes eight cytochrome P450-mediated oxygenations, five acetyl/aroyl transferase steps, and one epoxidation. Then, a reaction catalyzed by phenylalanine aminomutase (EC 5.4.3.10) takes place, which is followed by N-benzoylation and two coenzymes A esterifications. Together, paclitaxel biosynthesis from GGPP includes 19 enzymatic steps; it is reviewed in detail in [28].

The crucial step for the whole paclitaxel biosynthesis seems to be the cyclization of GGPP leading to taxadiene. Therefore, taxadiene synthase is the most studied enzyme involved in this pathway; thus, it is no wonder that the focus has been taken to enhance the activity of this enzyme so that the overall paclitaxel yield from yew is increased. Edgar et al. [29] confirmed that site-saturation mutagenesis of the active sites of taxadiene synthase leads to augmented paclitaxel production. The mutated taxadiene synthase provided 2.4 higher yields of paclitaxel precursor taxadiene-5α-ol; however, the biosynthetic pathway was probably altered unexpectedly, since an alternative product of the cyclization reaction was formed too, namely taxa-4(20)-11(12)-diene. As aforementioned, the increase in paclitaxel production demonstrated in this study was not immense; however, it is a confirmation of a certain improvement and additional rationally designed mutagenesis of this enzyme could probably lead to even higher paclitaxel yields.

However, as discussed above, since paclitaxel isolation from raw yew material is a very ecologically demanding process and production in other organisms is not cost-effective, total paclitaxel synthesis would be a vital option. Multiple such synthetic approaches have been described; however, none of them has been used commercially, so far [30,31,32,33,34,35,36,37,38,39,40,41]. Despite this lack of success, the effort in this research area continues and novel paclitaxel syntheses still emerge. The most recent synthesis of paclitaxel was reported by Kanda et al. [42], namely two-phase synthesis. Interestingly, Kanda et al. were inspired by the natural biosynthesis of paclitaxel unlike many previous approaches, which were designed rather retrosynthetically. Thus, this novel synthesis, similarly to biosynthesis, consists of two phases, the “cyclase” phase, and the “oxidase” phase. The biggest pitfall was represented by the oxidation phase, which naturally requires several complex synthetic steps. Synthesis under laboratory conditions relies on protecting other groups while oxidizing the chosen one, which, given the complexity of taxane structure, is an extremely challenging task. Because of the many side steps needed, the yield of this precisely optimized synthesis was only 0.0014%. Nevertheless, the idea to mimic the biosynthetic order of reactions is extraordinary among all syntheses presented to date; although, it seems that optimizing such a process would be a long run. Perhaps, some combination of retrosynthesis and cyclase phase synthesis (which gave satisfactory yields in Kanda’s study) might provide the desired effective method of taxane production.

Since total paclitaxel synthesis has remained only a fruitless effort, so far, several semisynthetic strategies have been developed to utilize most of the plant material, by which the overall yield of the extraction methods can be increased and the whole process can become less environmentally demanding. Ecology is a great question considering paclitaxel production, as for one kilogram of this compound, ca. 3000 yew trees are necessary using the standard extraction method of paclitaxel isolation from the yew bark. This represents an extreme ecological burden [43]. To avoid this, another method was developed by Bristol-Myers Squibb, nowadays the most commonly used method for the industrial production of paclitaxel, namely paclitaxel semisynthesis from its biosynthetic precursor 10-diacetyl baccatin III (10-DAB). 10-DAB resembles the chemical structure of paclitaxel, though it lacks a side chain at the C-13 position. This substance as well as paclitaxel is present in the crude extract of raw yew material (bark, needles, or branches) together with other taxanes. With the use of bacterial enzymes, it is possible to quantitatively convert the taxanes to 10-DAB. More specifically, C-13 taxolase and C-10 deacetylase have been isolated from a Gram-positive bacteria *Nocardioides albus* SC 13911, by which it is possible to convert practically 100% of the taxanes to 10-DAB. Besides, other taxoid compounds contained also in the crude extract including xylosyltaxanes can be transformed to 10-DAB using xylosidase from *Moraxella sp.* The C13 side chain, which is in the paclitaxel biosynthesis added to 10-DAB, can be also produced enzymatically [44]. In summary, utilizing 10-DAB for paclitaxel semisynthesis is convenient not only for its more eco-friendly method of preparation, higher product yields, and cost-efficiency, but it has also another advantage, and it is that 10-DAB is contained also in yew needles in relatively satisfying yields. Utilizing 10-DAB from the tree needles is beneficial, since they regrow and, thus, harvesting of whole trees is not necessary which results in less impact on the environment [45].

### 1.3. Mechanism of Action of Paclitaxel

**Figure 7:**
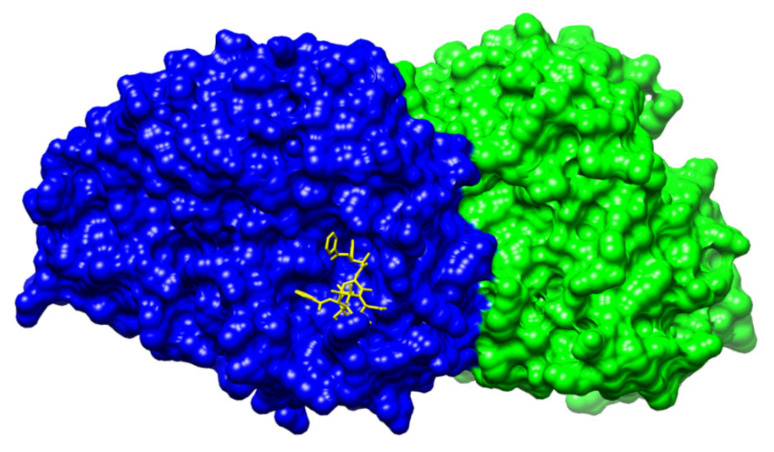
The binding site of paclitaxel on tubulin dimer, PDB (Protein Data Bank) structure 1jff adapted by Chimera software.

Soon after the first isolation of pure paclitaxel, its mechanism of action was determined. Paclitaxel is a tubulin-binding compound, which promotes the assembly of tubulin dimers and stabilizes microtubule fibers, and, thereby, arrests the cell cycle in mitosis. These properties make paclitaxel an ideal drug for cancer therapy, for which it is also broadly used. However, since immense research on taxanes has been conducted over the years, it is clear now that the high complexity of the antitumor action of taxanes has to be considered. Initial findings on paclitaxel as a cancer cure were rather naive. It was Wani et al. [1] who directly after paclitaxel isolation discovered its potent activity against various types of cancer. Eight years later, Schiff et al. [46] confirmed the ability of this compound to promote the assembly of microtubules, which are then resistant to depolymerization by either cold or CaCl_2_. During such assembly, the fibers formed are shorter and their amount in the cell is increased in comparison to physiological conditions. Soon after these findings, the ability of paclitaxel to bind to the compact microtubule fibers was proven [47]. The exact tubulin binding site was explored in 1992 and localized at the β-tubulin subunit (Figure 7) [48]. Since tubulin is a guanosine nucleotide-regulated protein and in its active state it is bound to guanosine triphosphate, the key discovery was made in 1992, when paclitaxel was proven to promote assembly of otherwise inactive tubulin bound to guanosine diphosphate. This explains the enhanced microtubule polymerization caused by this compound [49].

**Figure 8:**
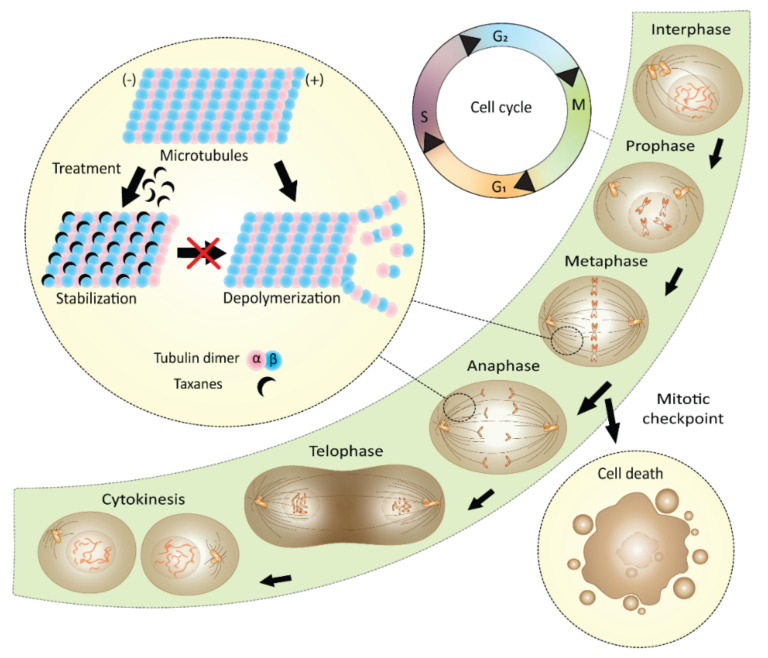
The mechanism of action of paclitaxel.

In mitotic cells, taxanes prevent microtubule attachment to the kinetochores. The unattached kinetochores cause mitotic arrest and the cell undergoes mitotic catastrophe, which leads to its death [50] (Figure 8), which can occur by different mechanisms. Taxanes trigger several molecular pathways: (i) induce phosphorylation of the anti-apoptotic factor B-cell lymphoma 2 (Bcl-2) [51], (ii) increase production of the cellular tumor antigen p53 (p53) even at very low concentrations not affecting the cell cycle [52], and (iii) induce endoplasmic reticulum stress. In connection to the last point mentioned, increased levels of certain factors, such as caspase 3 and 4 or C/EBP homologous protein, have been described in paclitaxel-treated cells. Endoplasmic reticulum stress plays an important role in cancer chemotherapy-induced neurotoxicity, which is an unpleasant side effect often occurring after treatment [53].

The mechanism of paclitaxel action is strongly dose-dependent. While at lower nanomolar concentrations (5–10 nM) mitosis is delayed, cells are still able to exit this state, even often with misaligned chromosomes. Treatment with higher paclitaxel concentrations (ca. to 10–500 nM) leads to prolonged mitotic arrest, the formation of altered mitotic spindles, and specific microtubule asters. These structures, which are not always nucleated by a centrosome, prevent normal mitosis and cells undergo apoptosis [54]. Microtubule asters are in some characteristics like spindles, at least by the presence of centrosomal material and calmodulin. Interestingly, asters do not grow from some organizing centers, but they are formed by the reorganization of microtubules, in which dyneins play a key role because microtubules slide along each other during this process [55]. The mechanism of paclitaxel action is more complex since the effect of the drug might be influenced by differences in the cell structure composition, for example, the microtubules.

Since there is not a single β-tubulin, but, eight β-tubulin subtypes have been described, a logical question arises: Does paclitaxel interact with all the tubulin subtypes similarly? The answer is no. It seems that exactly this aspect might play a decisive role in cancer cell resistance to paclitaxel. Particularly, the overexpression of class III β-tubulin (TUBB3) has been long considered as a possible cause of decreased cell sensitivity to paclitaxel. Öztop et al. [56] described TUBB3 as a potential predictive biomarker for the treatment of colorectal cancer with paclitaxel. The results of their study confirm that cancer cell sensitivity to paclitaxel treatment is dependent on TUBB3 levels since chemoresistant cells overexpressed this type of tubulin. However, there are also studies contradicting these results. Tame et al. [57] studied chemoresistant cancer cell line (RPE-20) which overexpresses TUBB3; however, they found out that this resistance is rather linked to the overexpression of the P-glycoprotein (P-gp) efflux pump. Experimenting with TUBB3 levels by affecting gene transcription did not show significant differences in paclitaxel action. Both findings denying each other only confirm the complexity of cell resistance to taxanes, which will be discussed in detail in Section 1.5.

### 1.4. Taxane Formulations for Improved Solubility and Tumor Delivery

Not only does cancer cell resistance represent a certain difficulty in taxane chemotherapy, but the problems also occur during the administration of taxanes due to their low water solubility. A lot of approaches to how to surmount this obstacle have been developed so far. In Taxol^®^, the most common clinically available formulation is paclitaxel diluted in a nonionic solubilizer and emulsifier called Cremophor^®^ EL (polyethoxylated castor oil) mixed with dehydrated ethanol (49.7%, *v*/*v*). Even though Cremophor^®^ EL markedly improves the paclitaxel solubility and has other positives, such as inhibition of P-gp in vitro and in vivo, it has also significant drawbacks, since it causes severe side effects such as hypersensitivity reactions leading to anaphylactic shock, neutropenia, and thrombocytopenia [58]. Another paclitaxel formulation includes Taxotere^®^, which is also commonly used in clinics. Nevertheless, similarly to Cremophor^®^ EL, Taxotere^®^ also exerts many undesired side effects since it also contains a nonionic surfactant and emulsifier, in this case, Tween^®^ 80 [59]. Despite the non-optimal performance of these taxane formulations, both are still broadly used in clinical practice, since only a few novel formulations passed clinical trials and reached commercially profitable production.

One of these is Abraxane^®^ (nab-paclitaxel), a nanoparticle albumin-bound formulation of paclitaxel, which received clinical approval from the FDA in 2005. This form of paclitaxel is also known as protein-bound paclitaxel. Abraxane^®^ has been used in the treatment of breast and non-small cell lung cancer and achieved better treatment outcomes than Taxol^®^. These findings do not exactly correspond to the levels of the compounds in blood plasma after administration, as one would expect. The concentration of Abraxane^®^ in blood plasma is three to five-fold lower than that of Taxol^®^ and yet the antitumor effect of Abraxane^®^ is stronger. As recently discovered, it could be explained by the ability of both compounds to affect cancer stem cells (CSCs) regulating the growth and progression of the whole tumor. However, each of the compounds affects CSCs differently. While Abraxane^®^ can decrease the frequency of CSCs in the breast cancer xenografts in nonobese diabetic/severe combined immunodeficiency mice, given most probably by increased penetration of this formulation into tumors; Taxol^®^ causes its increase [60]. The mechanism of such action of Taxol^®^ has remained unexplored, so far.

#### 1.4.1. Liposomes and Polymeric Micelles Containing Taxanes

Besides binding to albumin or using diverse chemical surfactants, there is another option how to increase taxane water solubility, namely their encapsulation into liposomes or micelles (Table 1). The first commercialized liposomal formulation of paclitaxel, the lipid core which contains lecithin and cholesterol, is Lipusu^®^ (Luye Pharmaceutical Co. Ltd., Shanghai, China), which has been clinically approved in China in 2006 for the treatment of ovarian, breast, and non-small cell lung carcinoma. Contrary to Taxol^®^, Lipusu^®^ does not induce anaphylactic shock and exhibits great distribution in practically all organs [61,62]. However, considering the high toxicity of paclitaxel, the rapid distribution into all organs might be linked with undesired side effects. Nevertheless, the advantages of the Lipusu^®^ agent surely prevail, showing liposomes to be the right way for paclitaxel (or other taxanes) delivery. Besides this already clinically approved liposomal paclitaxel formulation, other ones are yet clinically tested with distinct compositions. A so-called LEP-ETU (liposome-encapsulated paclitaxel—easy to use) marketed by Insys Therapeutics Inc. (Chandler, USA) has been granted orphan drug designation from the FDA to treat ovarian cancer [63]. The liposomes are in this case formed by dioleoyl-*sn*-glycero-3-phosphocholine, cholesterol, and cardiolipin [64]. This formulation passed phase II clinical trials on breast cancer. Further, EndoTAG-1 (Medigene AG, Planegg, Germany), a cationic liposomal (dioleoyl-3-trimethylammonium propane/dioleoyl-sn-glycero-3-phosphocholine) formulation of paclitaxel, which is targeted at negatively charged molecules on the surface of newly formed endothelial cells, is undergoing phase III clinical trials on pancreatic and breast cancer. This formulation exerts antiangiogenic properties mainly against tumor vasculature, where novel vessels are formed rapidly [65]. Last but not least, protein-free liposomal paclitaxel formulation PTX-LDE (liposome containing phosphatidylcholine, cholesterol and triolein), which mimics low-density lipoprotein particles, proceeded in phase II trials on epithelial ovarian carcinoma and displayed no cytotoxicity, indicating that the standard dose (175 mg·m^−2^) has to be increased. However, considering progression-free survival, which was in four patients higher than six months and in two patients more than one year, PTX-LDE offers a useful option for treating epithelial ovarian carcinoma.

Similar to paclitaxel, liposomal formulations of docetaxel have also been prepared, four of which have already been evaluated in clinical trials. Namely, LE-DT (NeoPharm, Inc., Waukegan, USA), with lipid particles of similar composition to that of LEP-ETU with added alpha-tocopheryl acid succinate, was well tolerated in patients with solid tumors with only mild side effects [75]. Similarly, ATI-1123 (Azaya Therapeutics, Inc., San Antonio, USA)—docetaxel in liposomal particles composed of phospholipids, cholesterol, human serum albumin, and saccharose, was tolerated by patients with solid tumors and has been recommended for phase II clinical trial [76]. Another liposomal formulation including docetaxel, MM-310 (Merrimack Pharmaceuticals, Inc., Cambridge, USA) ended up in phase I clinical trial exerting a low therapeutic index-linked mainly to cumulative peripheral neuropathy [77]. Finally, phase I study of another liposomal docetaxel formulation MNK-010 was terminated with no results published to date [78]. Despite such partial failures, liposomal formulations of taxanes represent a modern effective way of delivery of these anticancer drugs to tumors and offer the space for further research.

Recently, an interesting form of administration of liposomal paclitaxel formulation has been described. Namely, it is bacterial inhalation therapy for the treatment of lung cancer. The use of bacteria is an emerging approach in anticancer therapy because the tumor environment provides good conditions for bacterial growth, i.e., tumor necrosis provides nutrients and hypoxia decreases the action of macrophages and overall bactericidal processes. Zhang et al. [79] transformed *Escherichia coli* and *Lactobacillus casei* by the liposomal formulation of paclitaxel using electroporation. They confirmed that this formulation causes the greatest synergistic effect in comparison to the administration of liposomal paclitaxel and bacteria separately. That indicates that the use of bacterial inhalation therapy might be a promising direction in cancer research and therapy when combined with liposomal paclitaxel. Thus, liposomes as delivery media, either alone or in further combinations, have proved the usefulness for the delivery of taxanes into tumors. Moreover, they can also be used for the co-delivery of taxanes with other drugs, see in detail in Section 1.6.

Besides liposomes, taxanes can be also incorporated into polymeric micelles. Several such formulations have been already used in clinics. In South Korea, paclitaxel has been approved in a form of polymeric micelles called Genexol-PM^®^ (Cynviloq™, Samyang Corporation, Seoul, Korea) for the treatment of non-small cell lung carcinoma. The structural basis of these micelles is a low molecular-weight amphiphilic diblock copolymer monomethoxy poly(ethylene glycol)-block-poly(D,L-lactide) (mPEG-PDLLA). In addition, docetaxel is being used in the same formulation in polymeric micelles of the same copolymer system as Genexol-PM^®^, just marketed as Nanoxel^®^ (Samyang Corporation). Although this formulation has been currently commercialized, it seems that it altered the toxicological properties of docetaxel and, thus, it is necessary to elaborate it further and optimize its composition [80]. Another polymeric formulation of paclitaxel that received clinical approval is Paclical^®^ (Oasmia Pharmaceutical AB, Uppsala, Sweden). Under this brand name, it was clinically approved firstly in Kazakhstan but nowadays it is also available on the European market traded as Apealea^®^ (paclitaxel micellar). This formulation contains a surfactant XR17 which is a mixture of two isoforms of N-retinoyl-L-cysteic acid methyl ester sodium salt, retinoid derivatives [81]. The same formulation as Paclical^®^, only for use in dogs, is marketed under the brand Paccal Vet^®^.

#### 1.4.2. Hydrogel Formulations of Taxanes

In addition to liposomes or polymeric micelles, hydrogel formulations of taxanes were developed. Hydrogels are also formed by polymers, which create a three-dimensional hydrophobic network able to absorb a large amount of liquid and, thus, resemble a biological tissue. One of the greatest advantages of hydrogels is the ability to form a gel *in situ* at physiological conditions, either with the use of UV radiation or by chemical reactions, thus, avoiding usage of toxic polymerizing agents [82] Hydrogels can incorporate different drugs including taxanes and can be used for controlled local delivery of these drugs in tumors. For this purpose, chitosan-based hydrogel delivering paclitaxel was synthesized. A chitosan solution neutralized with a polyol counterionic dibase salt β-glycerophosphate is thermosensitive; it gelatinizes at body temperature. The mixture with paclitaxel was prepared simply by pouring the chitosan solution on the sterile drug powder and stirring for 4 h before salt was added to it. Such formulations injected into a tumor, exert an antitumor effect equal to four intravenous injections of Taxol^®^ in mouse models with implanted murine mammary carcinoma cells (EMT-6). Such effect, however, might not be linked only to paclitaxel, given the fact that chitosan alone has anticancer activity, or that direct intratumoral injection has to be logically more effective drug delivery [83]. With a similar effect, the chitosan hydrogel system can be used also for the delivery of docetaxel [84]. What makes hydrogels potentially very interesting for cancer treatment is their controlled sequential release of taxanes in the tumor. This fact could be advantageous for intraperitoneal chemotherapy, where a certain drug concentration during an extended period is needed. However, although Bajaj et al. 2012 [85] confirmed the increased retention of paclitaxel in the peritoneal cavity when delivered in hyaluronic acid-based gel, the anticancer activity was not improved. This might be caused by decreased solubility of the drug in the peritoneal environment. However, it is also possible to incorporate more water-soluble liposomes or micelles with paclitaxel into hydrogels. For example, Mao et al. [86] confirmed the functionality of paclitaxel-loaded liposomes in gel in vitro and in vivo in mice with inoculated S180 ascites sarcoma cells. Such formulation offers a good balance between anticancer effect and systemic toxicity, as liposomes are released more slowly from the gel and retained in the tumor for a prolonged time. As described, novel formulations of taxanes significantly improve the overall anticancer effect in comparison to standard clinically used Taxol^®^. In addition to the aforementioned approaches, there is another large group of taxane formulations designed for targeted delivery to tumors.

#### 1.4.3. Formulations for Targeted Delivery of Taxanes

Targeted delivery of anticancer drugs specifically into tumors substantially decreases the occurrence of side effects, which are common to many chemotherapeutics. To date, countless approaches for improved targeted taxane delivery have been invented. Surely, one of the most effortless ways how to target taxanes into tumors is to modify nanoparticles, which carry the drug. Delivery of taxanes in nanoparticles is a proven method, which is used already in clinics as described above on albumin-bound paclitaxel, Abraxane^®^. Besides albumin nanoparticles, formulations with poly(lactic-co-glycolic acid) (PLGA) are very popular, since this system is biodegraded to nontoxic products by hydrolysis. Similarly, for their biodegradability, poly(L-lactide), poly(butyl cyanoacrylate), or hyaluronic acid nanoparticles are widely used to deliver pharmacological compounds including taxanes. For a comprehensive review of nanoparticle taxane delivery, see ref. [87]. All these nanoparticle systems are currently being improved to reach the optimal distribution into tumors. One example for all might be a recent study of lipid-PLGA nanoparticles with human serum albumin as surfactant published by Godara et al. 2020 [88]. These nanoparticles combine PLGA as the polymer basis, stearyl amine, and soya lecithin as lipids ensuring slower polymer degradation by restraining inward diffusion of water and albumin as protein ensuring higher blood circulation because of the reduction in phagocytosis. The results of this study confirm these complementary effects and, thus, this formulation represents a potentially useful paclitaxel delivery system. Nanoparticles offer a great area for derivatization, thus providing options to incorporate molecules targeting into tumors.

Certain receptor-targeting molecules can be attached on the particle surface, e.g., antibody fragment against the particular receptor, which is overexpressed on the cancer cell surface. For example, in breast cancer cells, this can be the human epidermal growth factor receptor 2/proto-oncogene Neu (HER2/neu) [89], which is often overexpressed similarly to the estrogen receptor. The targeting of the estrogen receptor can be achieved by its natural ligand estrone bound to paclitaxel-loaded nanoparticles targeted to a tumor [90]. Nanoparticles can be further linked to any molecule of desire exhibiting a high affinity to some cancer markers. Mostly, such molecules are short peptides. One of the most recent examples is described by Ullah et al. 2020. They synthesized paclitaxel-loaded PLGA nanoparticles linked with arginyl–glycyl–aspartic tripeptide targeting glioblastoma, which can be administered intranasally and deliver paclitaxel directly nose-to-brain into glioblastoma cells unaffecting healthy brain cells. Another option is to use aptamers, short oligonucleotides (or also peptides) specifically binding molecular targets, illustrated for example on anti-heparanase aptamer-functionalized PLGA–paclitaxel nanoparticles targeting breast cancer cells [91]. A recent emerging approach is to coat the whole nanoparticle with different membranes. These might be directly cancer cell membranes, such nanoparticles might be used for anticancer vaccinations, erythrocyte–platelet hybrid membranes prolonging circulation time of nanoparticles in blood, or macrophage membranes which were recently confirmed to be able to target nanoparticles into melanoma cells B16F10 both *in vitro* and *in vivo* [92].

#### 1.4.4. Derivatives of Taxanes for Improved Tumor Targeting

Apart from all formulations discussed above, there is another option of how to target taxanes into tumors, namely their direct chemical derivatization. Several sites have been identified as suitable for modifications, mainly on the cyclic core of taxanes. Modifications of the side-chain have been identified to lead to loss of activity, although particular modifications are also possible. The most optimal sites for derivatization are the hydroxyl group on the hexane ring, the acetyl group on the eight-membered ring, or the benzoyl group. Structure-activity relationships have been reviewed by Fang et al. [93]. The first experiments with targeting taxanes into tumors by derivatization were performed already in the 1990s. Safavy et al. [94] linked paclitaxel to a synthetic heptapeptide targeting the bombesin/gastrin-releasing peptide receptor. The conjugate was evaluated in cells derived from non-small cell lung carcinoma (NCI-H1299) reaching the half-minimal inhibitory concentration (IC_50_) of 2.5-times lower than that of paclitaxel after 96 h treatment. Moreover, this conjugate was proven to be readily water-soluble. Unfortunately, the study of Safavy does not say anything about cancer selectivity of the derivative, since no experiment was performed using noncancerous cells. Ndungu et al. [95] developed a paclitaxel conjugate targeting cancer cells overexpressing tissue factor. The conjugate consisted of paclitaxel connected to factor VIIa by a tripeptide D-Phe-L-Phe-L-Arg linker terminated either by methyl ketone or chloromethyl ketone. Factor VIIa plays a crucial role in the blood clotting process, which begins by its binding to tissue factors. Given the overexpression of tissue factors in some types of cancer cells, patients often face increased blood clotting and thrombosis risk. Nevertheless, the binding of factor VIIa to chloromethyl ketone inactivates the clotting activation properties of this factor, which is, however, still able to bind tissue factors. Thus, such a derivative may selectively bind to cancer cells and lower the thrombosis risk of cancer patients. The paclitaxel-D-Phe-L-Phe-L-Arg-methyl ketone-factor VIIa derivative, which is internalized into cells by endocytosis after binding to tissue factor, displayed two times greater anticancer activity against cells derived from mouth epidermal carcinoma (KB cell line) than paclitaxel alone. On the contrary, in human umbilical vein cells, which do not normally express tissue factor, only free paclitaxel was cytotoxic. This indicates that, without binding to tissue factors, the novel derivative does not exhibit any toxicity. This would be of great benefit in terms of diminishing paclitaxel adverse effects.

To date, multiple other approaches for taxane derivatization have been described, see ref. [96,97]. For example, linking paclitaxel to vitamins from the B group, as their receptors are often overexpressed on some cancer cells. For example, folate receptor (folate—vitamin B_9_) is expressed on cancer cells because of their higher need for folate for DNA synthesis. Similarly, it couples with biotin (vitamin B_7_), receptors of which are expressed even more than the folate ones. Vineberg et al. synthesized a biotin receptor-targeted taxoid compound linked with biotin through a self-immolative disulfide linker further connected to fluorescein to obtain a theranostic compound. This compound exerted 100-times greater cytotoxicity on biotin-receptor positive cells (murine ovarian carcinoma cell line ID8) in comparison to cells lacking this receptor (human lung fibroblast cell line WI38) [98]. Besides vitamins, it is possible to link taxanes to antibodies. A pilot study of such conjugates was performed by Ojima et al., who linked a taxoid derivative to a monoclonal antibody against human epidermal growth factor receptor (EGFR). This derivative was highly tumor-specific and in A431 epidermoid carcinoma, xenografts showed complete inhibition of tumor growth in all treated mice [99]. Recently, antibodies are used mainly in combination delivery with taxanes, which will be discussed in Section 1.6.

An exceptional formulation of paclitaxel, which is linked to combination therapy, is oral-paclitaxel with P-gp (see Section 1.5. 2 inhibitor HM30181A ([2-(2-{4-[2-(6,7-dimethoxy-3,4-dihydro-1H-isoquinolin-2-yl)-ethyl]-phenyl}-2H-tetrazol-5yl-4,5dimethoxyphenyl]amide). This combination, in form of capsules, is traded as Oraxol^®^ (Hanmi Pharmaceutical Co. Ltd., Seoul, Korea). Among all the aforementioned formulations, which are practically all administered intravenously or in a form of injections into tumors, this formulation is administered orally and, thus, it offers new horizons in taxane treatment mainly in terms of patients’ comfort. Oraxol^®^ is currently being tested in eight clinical trials, one phase III trial is active in patients with metastatic breast cancer, one active phase II trial in patients with diverse solid tumors, and other trials in phase I also exist [100]. Based on a few published results, Oraxol^®^ seems to be well tolerated. In gastric cancer patients, a favorable toxicity profile as second-line therapy was described [101]. Developing an oral formulation of taxanes generally is surely the right way, considering the comfort of patients needing long-term therapy. However, Oraxol^®^ is yet to be a pioneering formulation in this field and further studies of this compound will have to take place to improve the efficacy and to obtain a sufficient amount of data for clinical use.

To sum up, a lot of diverse formulations of taxanes have been developed to increase their water solubility, decrease their cytotoxicity, and enhance their targeting specificity into tumor cells. All to improve the overall efficacy of these cytotoxic compounds. While early developments worked with chemical emulsifiers, such as Cremophor^®^ EL, which causes undesired side effects, modern applications rely on nanotechnologies, polymeric engineering, and specific ligand-derived formulations. The novel options are slowly replacing the old ones in clinical practice; however, since clinical trials are often time-consuming, surfactant-based formulations are still broadly used. Surely, nanoformulations of taxanes have a great future, because they can be diversely modified on their surface, offering a place for targeted therapy, but also co-delivery with other drugs (Section 1.6). The most probable further course of taxane development lies in a mixture of the aforementioned techniques, involving surface-modified liposomal nanoparticles encapsulating potently cytotoxic derivatives of natural taxanes. What will be the biggest challenge in taxane research, though, is to overcome cancer cell resistance to these compounds, which is the factor complicating the current taxane-based therapy the most.

### 1.5. Mechanisms of Cancer Cell Resistance to Taxanes

Cancer cell resistance to taxanes may be inherent, involving natural expression of enzymes active mainly in taxane metabolism, cell efflux pumps, etc., or acquired, which occurs after long-term treatment of cells with a drug that had initially a desired effect. Generally, the main mechanisms of cancer cell resistance to taxanes represent: (i) altered apoptotic pathways (e.g., absence of certain proapoptotic proteins), (ii) overexpression of efflux pumps actively transporting a drug out of the cells, (iii) decreased drug uptake (e.g., a decreased level of receptors), (iv) changes in tubulin composition (Section 1.3), (v) hypoxic conditions and microRNAs (miRNAs) function.

1. One of the most commonly known apoptotic factors linked to cancer cell resistance to paclitaxel is Bcl-2 protein, a proton-efflux pump acting as a pro-survival factor. Interestingly, this protein contains a paclitaxel-binding domain like in the β-tubulin and, therefore, it is strongly bound by paclitaxel. For sure, in nature, nothing is just a coincidence. Therefore, a naturally occurring ligand of both of these proteins must exist. Interestingly, this ligand has already been identified and it is the nerve growth factor IB (Nur77 or also NGFIB). Nur77 physiologically participates in cell death signaling by translocation from the nucleus to mitochondria and binding to Bcl-2, thus changing its properties from anti-apoptotic to pro-apoptotic [102]. Given the anti-apoptotic properties of Bcl-2, upregulation of this factor is often linked to cancer cell resistance to paclitaxel. To overcome this resistance, Wang et al. [103] suggested a co-delivery of paclitaxel with plasmid deoxyribonucleic acid (pDNA) for Nur77 in cationic polymeric micelles. This approach significantly increased the sensitivity of resistant cancer cell lines to taxane treatment. Besides Nur77, there are also further possibilities to regulate the Bcl-2 family of proteins, reviewed in ref. [104]. Bcl-2 proteins are not the only pump proteins playing a role in cell resistance to taxanes.
2. A significant contribution to cancer cell resistance to taxanes is made by drug efflux pumps, which are responsible for decreasing the effective concentration of a drug in a cell. One of the most common pumps is P-gp, which belongs to the ATP-binding cassette (ABC) transporters. Proteins such as P-gp are commonly overexpressed in multidrug-resistant cancer cells. So far, several approaches to avoid cell efflux of paclitaxel have been explored. Mainly, the option of paclitaxel administration in combination with diverse P-gp inhibitors such as quinines, verapamil, and curcumin have been examined [105,106,107]. The subgroup of ABCs mostly involved in cancer cell resistance to taxanes is ABCB1. Zhong et al. [108] suggested that ABCB1 polymorphisms could be used for predicting the effectiveness of taxane treatment. In their study on patients with non-small cell lung carcinoma, they identified two sites in the *ABCB1* gene, the mutations of which significantly correlated with enhanced cytotoxicity after taxane treatment. Generally, individuals with the wild-type genes had a longer progression-free survival rate. However, the study was conducted on a very specific and not very large group of Chinese patients and, therefore, it is not possible to make a general conclusion based on these results. Nevertheless, it has been shown that a correlation between the expression of particular ABC types and taxane treatment effectiveness exists; thus, further studies in this field are desirable.
3. Cancer cell resistance to taxanes may also consist of decreased cell uptake of the drugs. Taxanes, based on their hydrophobicity, enter cells rapidly via passive diffusion. Logically, changes in cell membrane composition may impede this process, mainly increased volume of cholesterol in the membrane. Taxanes also use specific transporters in the membranes to access cells. One of such transporters, organic anion transporting polypeptide 1B3, is often downregulated in chemoresistant cancer cells. Several chemoresistance mechanisms linked to decreased drug uptake were described in the case of paclitaxel nanoformulations, see Section 1.6. The lipophilic nanoformulations use the endocytic pathway to move paclitaxel into cells; thus, malfunctions of endocytosis may influence paclitaxel’s ability to enter cells. For example, modifications of the actin protein playing a crucial role in micropinocytosis, often correlating with cell resistance to taxane treatment. Besides actin, plastin-3 is another protein playing a role in endocytosis. Its downregulation significantly increases paclitaxel sensitivity. Cell chemoresistance caused by decreased drug uptake might be overcome by derivatization of the drug or by creating novel formulations. For example, mimicking low-density lipoproteins, as cholesterol-bound paclitaxel nanocarriers, might be a possible way to go [109].
4. An interesting cause of cancer cell resistance to taxane treatment is hypoxia. Hypoxic microenvironments are present in solid tumors, in which there is a reduced amount of blood vessels since tumor growth does not always correlate with the level of angiogenesis. When administered intravenously, the lack of vessels means that the opportunity for the drug to enter the tumor site is significantly reduced. At the same time, the rapidly proliferating tumor cells lacking blood vessels are promptly depleted in oxygen. Hypoxia triggers several signaling pathways leading to cell chemoresistance. The starting process of these pathways is the translocation of hypoxia-inducible factor 1 (HIF-1) into the cell nucleus and subsequent dimerization and, thus, induction of expression of genes involved in hypoxic response. Several studies confirm that silencing the gene expression of HIF-1 in cancer cells increases cell sensitivity to paclitaxel. Besides HIF-1 regulated pathways, during the hypoxic response, for example, the aforementioned ABCB1 is also overexpressed, or autophagy, which inhibits paclitaxel activity, occurs, illustrating the complexity of hypoxia’s contribution to cell resistance to taxanes. Zeng et al. also reported a certain correlation between HIF levels and changes in the morphology of tubulin. That would explain the increased resistance to paclitaxel in HIF-expressing cells [110,111,112].
5. Recent studies reveal that miRNAs play also a noticeable role in cell resistance to taxanes. The cellular function of miRNAs is to regulate gene expression by binding to messenger-RNAs (mRNAs). The most recent study discussing their role in cell resistance to taxanes was performed by Chen et al. [113], who identified two miRNAs, miR-335-5p and hsa-let-7c-5p, and their gene targets, chemokine (C-X-C motif) ligand 9, C-C chemokine receptor type 7, and suppressor of cytokine signaling 1, which are all linked to cell resistance to taxanes. Certain miRNA profiles are typical for paclitaxel-sensitive or chemoresistant tumors; these molecules could be used in the initial screening preceding the personalized treatment of cancer patients [114].

Although a lot of particular mechanisms of cell resistance to taxanes have been identified, overcoming this resistance is still not easy. Mainly it is because of combinations of these mechanisms in resistant cells. Therefore, it would be great if we were able to know which types of resistance factors are present in the case of the particular patient before the treatment. That is what personalized medicine focuses on and it is great that this approach is emerging more and more in cancer treatment. Once the resistance type is identified, we would choose the right treatment, in this case mainly the combinatorial treatment [115].

### 1.6. Combination Therapy with Taxanes

**Figure 9:**
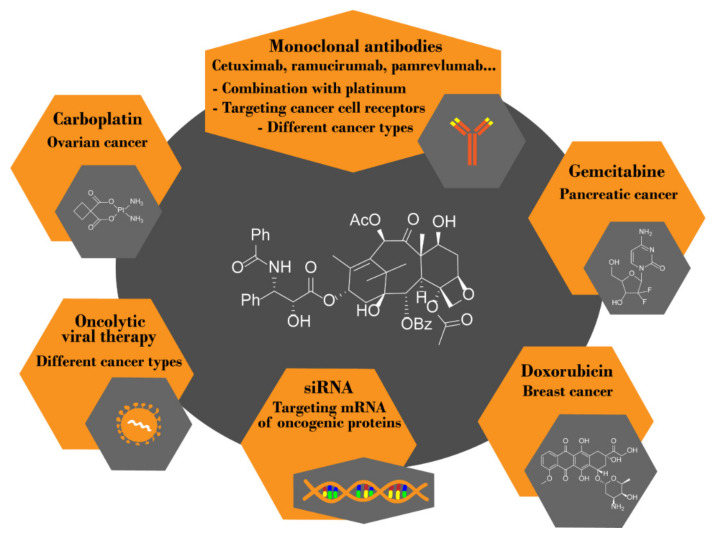
Chemical structure of paclitaxel, diverse approaches of combinatorial treatment with paclitaxel, and indications for particular drug combinations.

To increase the overall efficacy of cancer treatment, taxanes have started to be co-administered with other agents in so-called combination therapy; see the scheme of potential drug combinations for individual indications in Figure 9. This approach is used mainly to overcome cancer cell resistance to taxanes; therefore, taxanes are co-administered either with other chemotherapeutics to reach synergistic effect or combined with molecules reducing taxane adverse effect. As Taxol^®^ is used mainly for the treatment of ovarian, cervical, and breast cancer, it is no wonder that combinations with chemotherapeutics standardly applied for these types of carcinomas were the first choice. More specifically, cisplatin, being among the most effective anticancer drugs, so far, has been co-administered with paclitaxel for a long time. Since 1996, paclitaxel has been approved in combination with cisplatin for the first-line treatment of ovarian cancer and subsequent therapy of advanced ovarian cancer [116]. However, cisplatin itself is a relatively toxic substance; therefore, a less toxic, but similarly active, carboplatin has been introduced and is gradually replacing cisplatin in cancer therapy. The combination of carboplatin and Taxol^®^ was even given a special nickname “carbotaxol”. Currently, the “carbotaxol” combination is a broadly studied option for the treatment of diverse solid tumors. To date, there are 419 ongoing (active, recruiting, not yet recruiting, enrolling by invitation) clinical trials of this drug combination on cancer [117]. One of the most recent studies in this field reported by Safra et al. [118] deals with the scheduling of carboplatin/paclitaxel treatment cycles in first-line therapy of ovarian cancer. They found that administering a lower dosage (80 mg·m^−2^) of paclitaxel combined with AUC2 (area under the curve, dosing parameter meaning concentration of the drug in plasma in time) carboplatin weekly, instead of standard dosage (175 mg·m^−2^ paclitaxel plus AUC6 carboplatin) once in three weeks, increase the efficacy of such treatment and it is also better tolerated by patients. They confirmed this type of scheduling to be suitable for elderly and more susceptible patients with ovarian cancer.

#### 1.6.1. Combination of Paclitaxel and Immunotherapy

It is no exception, especially in cancer treatment, that in combination therapy more than two drugs can be co-administered. This holds also for platinum-based paclitaxel therapy of cancer since these drugs can be further combined with immunotherapy (mAbs)—see Table 2. In 2006, the FDA approved the combination of paclitaxel, carboplatin, and bevacizumab (Avastin^®^, Roche, Basel, Switzerland), a recombinant humanized monoclonal IgG1 antibody selectively binding and neutralizing the human vascular endothelial growth factor (VEGF), for the first-line therapy of advanced nonsquamous non-small-cell lung carcinoma [119]. In 2018, bevacizumab was further approved by the FDA for the treatment of advanced ovarian carcinoma. Although this drug combination is being commonly used today, there is still evidence that some patients do not respond to the therapy so well; moreover, severe side effects during the therapy were described. Therefore, much effort has been made to identify specific biomarkers linked to a patient’s response to a particular treatment. Recently, interleukin-6 (IL-6) was identified as a biomarker of efficiency of treatment with bevacizumab co-administered with paclitaxel and carboplatin. The higher the levels of IL-6 in a patient’s blood plasma, the better the prognosis of the treatment [120]. Bevacizumab is not the only mAb that can be co-administered with paclitaxel and carboplatin. A wide range of mAbs is currently being tested in clinical trials, see Table 2.

At present, combinatorial treatment of cancer with taxanes co-administered with immunotherapy using mAbs is an emerging approach, generally combining immunotherapy and chemotherapy (Table 3). This confirms also the fact that searching the terms “monoclonal antibody AND paclitaxel”, and “monoclonal antibody AND docetaxel” in ref. [129] gives 337 [130] and 143 [131] hits, respectively. One of the most successful mAbs for paclitaxel combination therapy is ramucirumab (Cyramza^TM^; Eli Lilly and Company, Indianapolis, USA). This drug combination received FDA approval in 2014 as a second-line treatment for advanced gastric carcinomas which progressed after the first-line chemotherapy [132]. Ramucirumab is a human monoclonal IgG1 antibody targeted against the VEGF receptor 2, the inhibition of which is a well-established method in cancer therapy. The results of phase III clinical trials of the paclitaxel combination with this anti-angiogenic agent were extremely encouraging. The study reported positive results on significantly enhanced overall survival or progression-free survival of patients recruited in the study while only minimum side effects (diarrhea, fatigue, or abdominal pain, plus adverse effects caused by taxanes themselves) were evidenced [133]. Recently, however, a case report of the development of fulminant Fournier’s gangrene in a patient treated with ramucirumab/paclitaxel, which does not correspond to changes in laboratory parameters, has been published. This gangrene may potentially lead to multiple organ failure. Nevertheless, this is just a single case report without any further research performed; thus, it is not clear whether the development of this gangrene was caused directly by the medication [134].

Since, generally, the ramucirumab/paclitaxel combination seems to be advantageous in gastric cancer treatment, the research of this drug combination is continuing. The current scientific question is whether it is better to use ramucirumab and paclitaxel as a first-choice treatment or as a replacement maintenance therapy. In 2019, the large ARMANI phase III clinical trial on evaluating the possibility to co-administer ramucirumab and paclitaxel after platinum/fluoropyrimidine unsuccessful first-line treatment has begun. No results have been published, so far [146]. Meanwhile, studies of the exact mechanism of the synergistic action of both drugs have been performed. Refolo et al. published results of the study of combinatorial treatment with paclitaxel and ramucirumab on four gastric cancer cell lines (HGC-27, NCI-N87 [N87], KATO III, and AGS) and reported ramucirumab to potentiate the growth inhibition caused by paclitaxel in all the cell lines. Levels of several molecular factors involved in the regulation of the cell cycle and cell death were altered after treatment with the drug combination; for example, certain cyclin-dependent kinases and cyclins. The ability of the drugs to induce apoptosis was confirmed to be higher when co-administered than when administered separately. Interestingly, ramucirumab and paclitaxel synergistically affected the mitogen-activated protein kinase and phosphatidylinositol 3-kinase/protein kinase B (PI3K/Akt) pathways. Although none of these drugs separately were able to modulate the phosphorylation levels of the proteins involved in this pathway, when co-administered, strong inhibition of protein phosphorylation was detected. To sum up, the synergistic effect of ramucirumab and paclitaxel co-administration demonstrated in this study was confirmed by all measured parameters, i.e., cell growth inhibition, cell cycle progression inhibition, expression of proteins crucial for cell motility, microtubule organization, or VEGFR expression [147]. Thus, it seems, that this combination of drugs might be a successful treatment approach for gastric cancer, though we have to wait for some statistically more relevant data concerning possible long-term side effects caused by this drug co-administration since it has been used clinically only since 2014.

Besides ramucirumab, recent studies suggest that paclitaxel co-administration with another mAb cetuximab could be a first-line therapy of squamous cell carcinoma of the head and neck in platinum-based chemoradiotherapy-refractory patients. Cetuximab is a chimeric mouse/human mAb binding EGFR in both cancerous and noncancerous cells. It competitively inhibits binding of the epidermal growth factor and tumor growth factor-alpha, by which it reduces tumor growth, and cancer cell migration and spreading. Antibody-dependent cellular cytotoxicity, the main mechanism of anticancer action of cetuximab, can be further increased by paclitaxel [148]. Co-administration of cetuximab and paclitaxel was reported to be effective not only for the treatment of head and neck carcinoma but also for cervical, esophageal, urothelial, or non-small cell lung carcinoma, on which there are currently active clinical trials [149,150,151,152,153,154,155,156,157,158,159,160,161,162,163]. Another recently reported promising combination of paclitaxel with immunotherapy involves pamrevlumab (FG-3019; FibroGen, Inc., San Francisco, USA). Pamrevlumab is a humanized monoclonal antibody inhibiting the action of the connective tissue growth factor, which is often overexpressed in cells from pancreatic ductal adenocarcinoma and plays a key role in the progression of this type of tumor. A randomized phase I/II trial on patients with localized advanced pancreatic cancer investigating the pamrevlumab and gemcitabine/nab-paclitaxel combination revealed that two times more patients were able to complete six treatments cycles compared to those treated solely with gemcitabine/nab-paclitaxel. Generally, the combination of these three drugs enhanced tumor response and increased tumor resection possibility, since normally pancreatic ductal carcinoma is impossible to resect because of encasement of mesenteric vessels by extensively deposited extracellular matrix. Given the encouraging results of the phase II trial, currently, the phase III clinical trial on this drug combination is ongoing [164].

Antibodies can also be used to reduce paclitaxel adverse effects, thus increasing treatment efficacy. One of such side effects is paclitaxel-induced peripheral neuropathy (PIPN), which occurs in 60–70% of cases [165]. To date, the molecular basis of PIPN has been quite well described and several antibody targets have been identified. One such example is matrix metalloproteinase 9 (EC 3.4.24.35), which can be inhibited by mAb against this enzyme, and thus PIPN can be prevented and reversed in CD-1 (cluster of differentiation 1) mice as reported by Tonello et al. [166]. On paclitaxel-treated mice, they also observed a decrease in neuroinflammatory factor levels after administration of the mAb. Since paclitaxel treatment potently increases the levels of these factors in blood plasma, direct impeding of the progression of neuroinflammatory pathways could also play a significant role in preventing PIPN. This was indeed confirmed by Huehnchen et al., who showed that, in IL-6 deficient mice, PIPN had not developed after paclitaxel administration. At the same time, pretreatment with IL-6-neutralizing mAb also prevented PIPN development [167]. IL-6 is certainly not the only factor involved in PIPN progression. A whole range of inflammatory cytokines play also a key role in this process, such as interleukin 20 (IL-20), which was even studied on a few healthy and gynecologic cancer patients. Chen et al. conclusively showed that an mAb against IL-20 is the right way to avoid PIPN [168]. As one can see, mAbs are generally very useful in terms of moderating the adverse effects of paclitaxel treatment; however, some of them have been identified to potentiate PIPN progression [169,170,171]. For example, aforementioned bevacizumab apparently promotes the neuropathy just when co-administered with paclitaxel. It is probably done by its impact on VEGFR, since VEGF which bevacizumab interferes with has certain neuroprotective properties [169].

#### 1.6.2. Combination of Paclitaxel with Diverse Low-Molecular Inhibitors

Combination therapy of cancer involving taxanes is not just about platinum or immunotherapy. There is a wide range of other substances that have been studied for this purpose. One option is combining taxanes with different low-molecular-weight inhibitors of various signaling pathways, growth factors, and their respective receptors. One such is gefitinib (Iressa; AstraZeneca, Cambridge, UK and Teva, Petach Tikva, Israel), a selective inhibitor of the EGFR tyrosine kinase. Combination of this substance with paclitaxel was approved by the FDA in 2015 for the treatment of metastatic non-small cell lung carcinoma [172]. Gefitinib has the same mechanism of action as erlotinib (Tarceva; Roche), another EGFR tyrosine kinase inhibitor. In the case of its combination with paclitaxel, however, phase III clinical trials in patients with advanced non-small-cell lung cancer did not show advantageous synergism [173]. Such a result is quite surprising though, given the fact that paclitaxel combination with mAbs targeted against EGFR has proven to be successful. Nevertheless, EGFR is not the only target inhibition of which could be beneficial in combination with paclitaxel treatment. Hu et al. prepared paclitaxel-loaded nanoparticles containing also indoleamine-2,3-dioxygenase inhibitor D-1-methyltryptophan. Indoleamine-2,3-dioxygenase (EC 1.13.11.52) is an immunosuppressive enzyme, which seems to be a promising target for cancer immunotherapy. Monotherapy with its inhibitors did not exert positive treatment results; however, when combined with paclitaxel, the outcome was significantly improved [174].

In addition to EGFR and indoleamine-2,3-dioxygenase inhibitors, another recently published option is a combination of paclitaxel with sorafenib (Nexavar; Bayer, Leverkusen, Germany and Onyx Pharmaceuticals, Newbury Park, USA), a commonly used chemotherapeutic, which is a tyrosine-kinase inhibitor affecting mainly the RAF/MEK/ERK pathway. Sorafenib was approved as a first-line treatment for advanced renal cell carcinoma (2005), unresectable hepatocellular carcinoma (2007), and metastatic differentiated thyroid cancer (2013) [175]. Nawara et al. [176] confirmed that a combination of sorafenib and paclitaxel at nontoxic concentrations of these drugs had substantially suppressed the oncogenic action of CSCs and, thereby, tumorigenesis progression. Such an approach could be beneficial in terms of reducing the side effects of common anticancer therapy. Furthermore, another low-molecular-weight inhibitor has been evaluated in combination with paclitaxel, namely, vismodegib (Erivedge; Genentech, South San Francisco, USA), the first FDA-approved inhibitor of the Hedgehog signaling pathway. The study on patients with metastatic pancreatic adenocarcinoma, unfortunately, did not show increased efficacy of this combination in comparison to commonly used therapy (gemcitabine + nab-paclitaxel) [177]. Another clinically studied combination of paclitaxel with a signaling pathway inhibitor involves everolimus (Afinitor, Votubia; Novartis, Basel, Switzerland), which blocks the mammalian target of rapamycin. This drug combination synergistically acts against a wide range of cancer types in vitro; however, recent clinical trials did not confirm the efficacy of this drug combination in vivo [178]. Therefore, the most recent studies are focused on improved delivery of both drugs, mainly by nanoparticles. One such example is nanoparticles targeted via Fab antibody fragments on HER2/neu and EGFR on breast cancer cells. This strategy has significantly improved the anticancer efficacy of paclitaxel/everolimus co-administration [89].

One of the very commonly used drugs for combination therapy of cancer involving paclitaxel is gemcitabine (Gemzar, Eli Lilly, and company). Gemcitabine is administered as a prodrug which after activation inhibits thymidylate synthase (EC 2.1.1.45)), thereby blocking DNA synthesis leading to cell death. Gemcitabine activation occurs at physiological conditions in the intracellular environment, in which deoxycytidine kinase converts this prodrug into two active metabolites—gemcitabine diphosphate and triphosphate [179]. The most often treated indication by this drug combination (gemcitabine and nab-paclitaxel) is advanced pancreatic cancer; this drug combination significantly increases the overall survival patient rate [180]. Recent studies mainly describe a potential co-administration of gemcitabine and paclitaxel also with other drugs, such as nivolumab, human IgG4 monoclonal antibody which blocks the immune checkpoint—programmed cell death protein 1 (CD-279), which sensitizes cancer cells for the immune system and causes the induction of immunogenic cell death. The most recent study on this triple combination evaluated for the treatment of pancreatic, breast, and non-small cell lung carcinoma in the first phase of clinical trials did not confirm recommend this drug combination for further studies, due to lack of clinical efficacy [181]. However, more clinical trials examining these three drugs in combination with other substances on pancreatic adenocarcinoma are currently being conducted [182].

Another very broadly used combination therapy of cancer is paclitaxel co-administered with doxorubicin. Doxorubicin is an anthracycline analogue base and its mechanism of action includes inhibition of topoisomerase II, intercalation into DNA, and induction of reactive oxygen species production. Mostly, this combination is used to treat breast cancer. To date, mainly improvements in the common delivery of both drugs have been studied, mainly concerning the use of nanoparticles [183]. Quite an interesting approach of doxorubicin and paclitaxel co-delivery to tumors was performed by Kaur et al., who developed an inhalable spray of dried nanoparticles loaded with both drugs. This formulation aimed for lung cancer treatment exhibited better distribution in the lungs than plain drugs and it could be one of the treatment options for this type of cancer [184]. However, nanoparticles in this particular study included the aforementioned Cremophor^®^ EL known for causing many adverse effects; therefore, it is questionable whether this formulation will pass the clinical trials. Furthermore, combinations of paclitaxel and doxorubicin with other drugs are possible. An interesting combination was shown in Fraguas-Sánchez et al., who prepared cannabidiol-loaded nanoparticles which were co-administered with paclitaxel and doxorubicin. Cannabidiol is well-known for its anticancer activity mainly against breast tumors and the combination with paclitaxel significantly exerted a synergistic effect [185].

#### 1.6.3. Other Therapeutical Combinations of Paclitaxel

Among modern therapeutic agents, short interfering RNAs (siRNAs) binding to mRNA represent a vital targeted anticancer approach. Since a lot of oncogenic proteins and factors have been identified so far, the approach of preventing the expression of these proteins is a logical way to combat cancer. Generally, siRNAs and paclitaxel are delivered in nanoparticles and lipophilic polymeric micelles. Zhu et al. performed *in vitro* co-delivery of VEGF-targeted siRNA and paclitaxel in cationic biodegradable micelles based on poly(2-(dimethylamino)ethyl methacrylate), which were shown to be able to efficiently transfect cancer cells with the siRNA and also deliver paclitaxel into cells. The synergistic action of VEGF inhibition and paclitaxel action results in increased cytotoxicity. The micelles themselves are practically non-toxic [186]. Besides anti-VEGF siRNA, experiments were performed with siRNA against key proteins in cancer, such as polo-like kinase 1 (serine-threonine kinase involved in the cell cycle) [187], aurora kinase A (serine-threonine kinase important for mitotic spindle formation) [188], induced myeloid leukemia cell differentiation protein (anti-apoptotic protein) [189] or survivin (a member of the family of inhibitors of apoptosis) [190]. The general success of all these combinations indicates that specific targeting of mRNA coding proteins involved in carcinogenesis is a promising approach and it is only a matter of time for these formulations of taxanes and siRNAs to be routinely used for cancer treatment.

Another option of how to efficiently eradicate tumor cells is to combine more mitotic poisons. Mitotic poisons do not generally target only microtubules, as taxanes do, but they target also other proteins playing an essential role in cancer, i.e., aurora and polo-like kinases and kinesins, summarized in ref. [191]. A combination of antimitotic drugs that proceeded already to clinical trials (five completed, one inactive phase), is paclitaxel co-administered with an inhibitor of aurora kinase A, alisertib [192]. Besides that, Yasuhira et al. combined paclitaxel *in vitro* with an inhibitor of kinesin-5, S-trityl-L-cysteine, which resulted in more than four times longer cell cycle arrest in mitosis than for paclitaxel alone [193]. A different approach of combining mitotic poisons was performed by Bombuwala et al., who connected paclitaxel directly to another antimitotic agent, colchicine. This conjugate called colchitaxel exerted antimicrotubule activity, which was not as potent as for the compounds administered in combination. The disorganization of microtubule fibers was only observed for extremely high concentrations of colchitaxel (higher than 12 µM) [194]. Besides the aforementioned drug combinations, there are surely many more different substances, the effect of which can enhance the anticancer activity of taxanes. One of them, which even proceeded to clinical trials, is curcumin, a natural phenolic compound known mainly for its nutritious properties. Curcumin has been used for a long time in traditional medicine since it exerts antioxidant, anti-inflammatory, renoprotective as well as anti-cancer properties; for more details, see ref. [195]. Its anticancer potential is probably driven by its pro-apoptotic activity triggering the PI3K/Akt pathway. Moreover, its potential to inhibit cell migration suppressing cancer cell metastasis was also described. When administered with paclitaxel, curcumin increases the efficacy of taxane treatment, which can be explained by curcumin’s potential to down-regulate P-gp expression, resulting in augmented paclitaxel concentration inside cancer cells. By affecting various signaling pathways, curcumin sensitizes the cells to paclitaxel therapy, based on which lower paclitaxel concentrations can be used and, thus, side effects are eliminated [196]. However, the medicinal benefits of curcumin are still questioned. Curcumin has for example a very poor bioavailability, indicating that its degradation products are responsible for the biological activity [197]. In addition, none of the double-blinded, placebo-controlled clinical trials of curcumin was successful [198].

Another approach for cancer treatment is the utilization of oncolytic viruses, which have already been approved by the FDA and, thus, are considered safe and effective for combination therapy. A combination of classic chemotherapy with oncolytic viruses is profitable since viruses act differently than chemotherapeutics, i.e., this synergy increases the anti-cancer effect. Formulation of paclitaxel and oncolytic adenovirus encapsulated in extracellular vesicles significantly enhanced anticancer activity both in vitro and in vivo in lung cancer models. Interestingly, combined with paclitaxel, transduction of the adenovirus was more successful than when delivered alone. The detailed molecular mechanism has not been revealed, yet. However, transcriptomic analysis shows certain changes in molecular factors within the cells; thus, a novel type of cellular response occurs after common delivery of paclitaxel and oncolytic virus in extracellular vesicles [199]. Lal et al. studied co-delivery of paclitaxel and oncolytic measles virus with incorporated human proapoptotic gene *BNIP3* coding BCL2/adenovirus E1B 19 kDa protein-interacting protein 3. Lal et al. confirmed the synergic effect in two breast cancer cell line models of MCF-7 and MDA-MB-231, the second of which represents a triple-negative breast cancer model. Caspase 3 levels were increased more in cells affected by the combination of drug and virus than in cells affected only by the virus. Moreover, a higher number of propidium iodide-stained cells occurred after the addition of paclitaxel to the virus-pretreated cells suggesting the synergic action [200]. To sum up, a combination of oncolytic virus therapy and taxanes seems to be an effective type of anticancer therapy and it proceeded even into clinical trials (Table 4).

Besides paclitaxel, other taxanes or next-generation toxoids are also currently being evaluated in clinical trials in combination therapy for cancer treatment. The general principles of possible drug combinations with other taxanes are similar to those of paclitaxel. For example, the term “docetaxel combination” gives 1265 results in the www.clinicaltrials.gov database (accessed on 21 January 2021) [201]. Here, we focus only on the most recent studies involving docetaxel combination therapy. Bishop et al. investigated combined administration of docetaxel with an inhibitor of tumor necrosis factor receptor-associated factor 6 (TRAF6). This combination might be beneficial in breast cancer metastasized in bones because TRAF6 is overexpressed on osteotropic breast cancer cells and even greater levels of this protein are detected in patients with bone metastasis [202]. Combinations with the same agents as aforementioned by paclitaxel are currently being studied also with docetaxel. Namely, combinations with doxorubicin, platinum, or monoclonal antibodies are being examined [203,204,205]. Furthermore, cabazitaxel may be combined with various agents. One of the most recent studies published in this field describes co-delivery of siRNA against triple-negative breast cancer oncogene *IKBKE* (Inhibitor of nuclear factor kappa-B kinase subunit epsilon) and cabazitaxel in nanocomplex modified with hyaluronic acid-targeting cancer cells. This combination represented a successful anticancer approach based on the synergistic action of both agents in vivo and also in vitro [206]. Similar to the aforementioned paclitaxel, cabazitaxel may be delivered in combination with curcumin. Chen et al. 2020 confirmed the synergism of both drugs when delivered in lipid–polymer nanoparticles [207].

## 2. Conclusions

Despite giant leaps made in cancer therapy over the last decades, taxanes remain one of the most clinically used groups of cancer therapeutics. It is due to their unique, strong, and very specific binding to tubulin, which causes cell cycle arrest and cell death. There are, however, certain limitations linked to these substances. First, the production of taxanes is an extreme ecological burden, since 10 tons of yew material are needed for 1 kg of paclitaxel [208]. Unfortunately, in comparison to novel ecologically suitable methods, such as biotechnological production, isolation from yew is still much more profitable. Second, there is a rising problem with cancer cells’ chemoresistance to taxanes. To overcome this problem, drug combinations including taxanes were developed and registered for cancer treatment, e.g., taxanes plus platinum, sundry low-molecular inhibitors, siRNAs, other mitotic poisons, or antibodies. Last but not least, taxanes are limited by their poor water solubility. Therefore, novel delivery systems and taxane formulations were developed and are already being marketed, mainly liposomal ones. In addition, other options, such as various nanoparticle-based systems and taxane derivatives are under development. A significant effort has been devoted to further tailoring of anticancer properties of taxanes since they are the first-line drugs for cancer therapy. This review article is proof of unceasing interest in these compounds. It is certain that among all cancer therapeutics used in clinics, taxanes will remain the leading compounds and novel approaches for their higher production will emerge.

## Figures and Tables

**Table 1:** Several completed and ongoing (active, recruiting, and not yet recruiting) clinical trials of novel nanosomal and micellar formulations of taxanes. Data from 22^nd^ of January 2021

Taxane Formulation	Number of Completed and Ongoing Clinical Trials				Ref.
	Completed	Active	Recruiting	Not Yet Recruiting	
Lipusu^®^	- ^1^	1	-	-	[66]
LEP-ETU	3	-	-	-	[67]
EndoTAG-1	-	4	2	-	[68]
PTX-LDE	-	-	-	-	[69]
LE-DT	2	-	-	-	[70]
ATI-1123	1	-	-	-	[71]
Genexol-PM^®^	8	-	1	-	[72]
Nanoxel^®^	-	-	1	1	[73]
Paclical^®^	1	-	-	-	[74]

^1^ no clinical trial registered.

**Table 2:** Monoclonal antibodies tested in combination with paclitaxel and carboplatin and their molecular targets.

Monoclonal Antibody	Trade Name	Company	Molecular Target	Ref.
Oregovomab	OvaRex^®^	Quest PharmaTech	Human mucin 16/cancer antigen 125	[121]
Lumretuzumab	n.t.	Roche	Human epidermal growth factor receptor 3	[122]
Nivolumab	Opdivo^®^	Bristol Myers Squibb	Programmed cell death 1 protein	[123,124]
Atezolizumab	Tecentriq^®^	Roche	Programmed death-ligand 1	[125]
Trastuzumab	Herceptin^®^	Roche	Human epidermal growth factor receptor 2	[126]
Pertuzumab	Perjeta^®^	Roche	Human epidermal growth factor receptor 2	[126]
Cetuximab	Erbitux^®^	Merck KGaA, Eli Lilly, Bristol-Myers Squibb	Epidermal growth factor receptor	[127]
Necitumumab	Portrazza^®^	Eli Lilly	Epidermal growth factor receptor	[128]

n.t.—does not have a trading name.

**Table 3:** Most recent ongoing clinical trials of paclitaxel in combination with other anticancer drugs (the latest phase of clinical trials, first posted in 2019 and 2020).

Compound Combination	Condition	Status	Phase	Clinical Trial Identifier	Ref.
Paclitaxel + carboplatin	Breast cancer	not yet recruiting	IV.	NCT04136782	[135]
Paclitaxel + carboplatin + bevacizumab	Non-small cell lung cancer	recruiting	III.	NCT04194203	[136]
		recruiting	III.	NCT04325698	[137]
		recruiting	III.	NCT04416035	[138]
Paclitaxel + carboplatin + bevacizumab + atezolizumab	Non-small cell lung cancer	recruiting	III.	NCT03991403	[139]
Paclitaxel + carboplatin + pembrolizumab	Squamous head and neck carcinoma	recruiting	IV.	NCT04489888	[140]
Paclitaxel + carboplatin + doxorubicin	Ovarian cancer	recruiting	IV.	NCT03794778	[141]
Paclitaxel + ramucirumab	Gastroesophageal cancer	not yet recruiting	III.	NCT04499924	[142]
Paclitaxel + cetuximab	Squamous head and neck carcinoma	recruiting	II.	NCT04278092	[143]
Paclitaxel/gemcitabine + pamrevlumab	Pancreatic cancer	recruiting	III.	NCT03941093	[144]
Paclitaxel + gemcitabine	Pancreatic cancer	unknown	IV.	NCT03401827	[145]

**Table 4:** Selected clinical trials for cancer treatment with paclitaxel in combination with other anticancer approaches.

Drug Combination	Search Term	Number of Clinical Trials (by October 2020)
Paclitaxel + carboplatin + bevacizumab	paclitaxel carboplatin bevacizumab	164
Paclitaxel + ramucirumab	paclitaxel ramucirumab	34
Paclitaxel + cetuximab	paclitaxel cetuximab	70
Paclitaxel + gefitinib	paclitaxel gefitinib	20
Paclitaxel + erlotinib	paclitaxel erlotinib	51
Paclitaxel + sorafenib	paclitaxel sorafenib	28
Paclitaxel + vismodegib	paclitaxel vismodegib	5
Paclitaxel + everolimus	paclitaxel everolimus	57
Paclitaxel + gemcitabine	paclitaxel gemcitabine	499
Paclitaxel + doxorubicin	paclitaxel rubicin	284
Paclitaxel + alisertib	paclitaxel alisertib	8
Paclitaxel + curcumin	paclitaxel curcumin	2
Paclitaxel + viral therapy	paclitaxel viral	41

## Data Availability

Not applicable.

## Abbreviation

10-DAB	10-deacetyl baccatin III
ABC	ATP-binding cassette transporters
AUC	Area under curve
Bcl-2	B-cell lymphoma 2
CD	Cluster of differenciation
CSC	cancer stem cells
DNA	deoxyribonucleic acid
EGFR	the epidermal growth factor receptor
EMA	European Medicines Agency
FDA	U.S. Food and Drug Administration
GGPP	geranylgeranyl pyrophosphate
HIF-1	hypoxia-inducible factor 1
IL-6	interleukin 6
IL-20	interleukin 20
mAbs	monoclonal antibodies
miRNAs	microRNAs
mPEG	poly(ethylene glycol) methyl ether
Nur77	nerve growth factor IB
p53	the cellular tumor antigen p53
P-gp	P-glycoprotein
PI3K/Akt	phosphatidylinositol 3-kinase/protein kinase B
PDLLA	monomethoxy poly(ethylene glycol)-block-poly(D,L-lactide)
PIPN	paclitaxel-induced peripheral neuropathy
PLGA	polylactic-co-glycolic acid
siRNA	short interfering RNAs
TRAF6	tumor necrosis factor receptor-associated factor 6
TUBB3	class III β-tubulin
VEGF	vascular endothelial growth factor
